# Optimizing Layer Thickness in Multi-Planar Volume Reconstruction for Distinguishing Invasive Adenocarcinoma from Non-Invasive and Minimally Invasive Lesions in Pulmonary Nodules (≤15 mm): A Comparative Study with Conventional Lung Window Settings

**DOI:** 10.3390/diagnostics16020220

**Published:** 2026-01-09

**Authors:** Ke Zhang, Wen-Tao Zhang, Ji-Wen Huo, Wei-Wei Jing, Si-Fan Chen, Mao-Lu Tan, Fa-Jin Lv

**Affiliations:** Department of Radiology, First Affiliated Hospital of Chongqing Medical University, 1 Youyi Rd., Yuanjiagang, Yuzhong, Chongqing 400016, China; zk18704558886@163.com (K.Z.); 15283669300@163.com (W.-T.Z.); hjw781222711@163.com (J.-W.H.); jweiwei2019@163.com (W.-W.J.); sifanchen20@hotmail.com (S.-F.C.); tanmaolu66@163.com (M.-L.T.)

**Keywords:** multi-planar volume reconstruction, layer thickness, invasive adenocarcinomas, differentiating diagnosis

## Abstract

**Objective:** To determine the optimal layer thickness for multi-planar volume reconstruction (MPVR) in differentiating invasive adenocarcinoma from non-invasive and minimally invasive lesions in pulmonary nodules (≤ 15 mm). **Materials and Methods:** This retrospective study enrolled a total of 601 solitary pulmonary nodules (≤15 mm) between June 2020 and February 2024, including 404 invasive adenocarcinomas (IAC), 80 micro-invasive adenocarcinomas (MIAs), 96 adenocarcinomas in situ (AISs), and 21 atypical adenomatous hyperplasias (AAHs). Thin-section computed tomography (TSCT) images with lung window settings and MPVR images with varying layer thicknesses (ranging from 2 to 14 mm with intervals of 2 mm) were analyzed for their morphological characteristics. Multivariate logistic regression analysis was employed to develop models for differentiating invasive adenocarcinoma from non-invasive and minimally invasive lesions. The model’s performances were further evaluated and compared to identify the optimal thickness for diagnosis. **Results:** The 10 mm MPVR model exhibited the best performance (AUC: 0.910, 95% CI [confidence interval]: 0.905–0.914; sensitivity: 0.906; specificity: 0.753; accuracy: 0.856; PPV: 0.883; and NPV: 0.796). As the MPVR layer thickness increased from 2 mm to 10 mm, model performance improved, with sensitivity rising from 0.870 to 0.906, specificity rising from 0.519 to 0.753, and accuracy increasing from 0.755 to 0.856. However, for layer thicknesses of 12 mm to 14 mm, all of them decreased. Furthermore, the overall performance of the 10 mm MPVR model surpassed that of the lung window model (AUC: 0.841, 95% CI: 0.831–0.844; sensitivity: 0.787; specificity: 0.760; accuracy: 0.778; PPV: 0.871; and NPV: 0.634). **Conclusions:** MPVR images with varying layer thicknesses can effectively distinguish invasive adenocarcinoma from non-invasive and minimally invasive lesions in pulmonary nodules ≤ 15 mm. Notably, the diagnostic performance of the 10 mm model was superior to model built with TSCT images, showing great potential as a precise and non-invasive tool for assessing the invasiveness of adenocarcinomas ≤ 15 mm.

## 1. Introduction

Lung cancer is the leading cause of cancer-related deaths globally. Adenocarcinomas account for approximately 35–40% of all lung cancer cases [1,2]. According to the current pathological classification, lung adenocarcinomas are categorized into preinvasive lesions (including atypical adenomatous hyperplasia (AAH) and adenocarcinoma in situ (AIS)), minimally invasive adenocarcinoma (MIA), and invasive adenocarcinoma (IAC) [3]. This histological stratification directly dictates clinical management and prognosis [4].

Accurate discrimination among these entities is therefore paramount but becomes particularly challenging in small (≤15 mm) solitary pulmonary nodules, which frequently lack definitive invasive features on conventional computed tomography (CT) [5,6]. This diagnostic difficulty is compounded by the fact that clinical management differs radically: whereas preinvasive and minimally invasive lesions are typically cured by sublobar resection with excellent long-term survival, IAC necessitates anatomic lobectomy and carries a risk of recurrence and metastasis [7,8,9]. Furthermore, this difficulty is especially pronounced in East Asian populations, who have a higher prevalence of indolent, early-stage adenocarcinomas among never smokers, a group in which traditional risk assessment tools based on smoking history are less effective.

Current strategies to improve IAC identification ≤ 15 mm include conventional CT morphology and radiomics. While radiomics enhances benign–malignant differentiation in screening cohorts [10], its clinical translation is limited by protocol-dependent reproducibility and inter-observer variability [11]. A notable gap in the literature is the insufficient evaluation of advanced three-dimensional post-processing techniques, such as multiplanar volume reconstruction (MPVR), for this specific diagnostic task [12,13,14]. Furthermore, existing research has largely focused on subsolid (ground-glass) nodules, while the potentially more consequential differentiation of invasiveness within solid nodules ≤ 15 mm remains less explored. Crucially, the impact of a key MPVR technical parameter—reconstruction slab thickness—on diagnostic performance for this purpose has not been established, representing a critical knowledge gap [15].

To address these gaps and formally evaluate the potential advantages of MPVR, we pre-specified the following primary and secondary hypotheses. Primary hypothesis (H1): MPVR image analysis provides superior diagnostic performance for distinguishing IAC from minimally invasive and preinvasive lesions (≤15 mm) compared to conventional thin-section computed tomography (TSCT) with lung window settings. Secondary hypotheses (H2–H4): (H2) The diagnostic performance of MPVR is dependent on reconstruction layer thickness, with an identifiable optimal thickness that maximizes the area under the receiver-operating characteristic curve (AUC); (H3) MPVR interpretation is associated with a shorter reading time than standard TSCT lung window reading while maintaining diagnostic accuracy; and (H4) MPVR improves the inter-observer agreement for key morphological feature assessment compared to TSCT.

## 2. Methods

### 2.1. Patients

**Figure 1:**
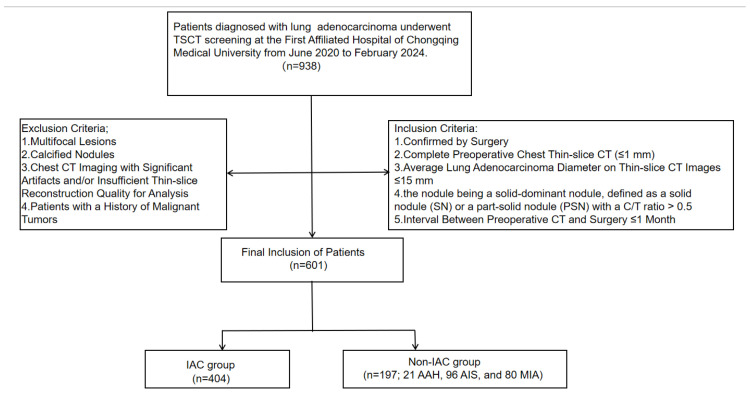
Flow chart of patient selection in this study.

This retrospective study was approved by our institution. Due to its retrospective nature, the requirement for informed patient consent was waived. The study included patients who underwent TSCT screening at our institution from June 2020 to February 2024 and were diagnosed with pulmonary adenocarcinoma. The inclusion criteria were as follows: (1) confirmed by surgery pathologically; (2) complete preoperative chest CT with a thin slice (≤1 mm); (3) average lung adenocarcinoma diameter on thin-slice CT images ≤ 15 mm; (4) the nodule being a solid-dominant nodule, defined as a solid nodule (SN) or a part-solid nodule (PSN) with a C/T ratio > 0.5; (5) the interval between preoperative CT and surgery is ≤ 1 month. The exclusion criteria were as follows: (1) multifocal lesions (to ensure each nodule represented an independent observation for statistical analysis); (2) calcified nodules; (3) chest CT imaging with significant artifacts and/or insufficient thin-slice reconstruction quality for analysis; and (4) patients with a history of malignant tumors. Ultimately, 601 patients with 601 nodules were enrolled and categorized into two groups: an IAC group (n = 404) and a non-IAC group comprising AAH, AIS, and MIA (n = 197; 21 AAH, 96 AIS, and 80 MIA), as illustrated in Figure 1.

### 2.2. CT Screening

All chest CT examinations were performed using a 128-MDCT scanner (Somatom Definition Flash; Siemens Healthineers, Erlangen, Germany) at the end of a single breath-hold inhalation. All CT acquisitions were scanned from the lung apex to the base. The imaging parameters were as follows: tube voltage of 110–120 kVp, tube current of 50–150 mAs, automatic exposure control technology, 0.5 s rotation time, 5/5 mm image slice thickness and interval, 64 × 0.625 mm detector collimation, 1.0 spacing; and a caudal–cranial scanning direction. All images were reconstructed with a slice thickness of 1 mm and a 512 × 512 matrix, using a lung algorithm for lung window images.

### 2.3. Image Interpretation

Standard lung window settings (width, 1600 Hounsfield units (HU); level, −600 HU) were used to independently evaluate images from the Picture Archive and Communication System (PACS) workstation (Vue PACS, Carestream, version 12.2.6.3000020). Observers were allowed to make moderate adjustments to the window settings to maintain normal workflow. To ensure reproducibility and minimize operator-dependent variability, all MPVRs were performed using fixed, pre-defined parameters. Specifically, the CT value threshold was set at −800 HU to differentiate pulmonary nodules and vessels from the lung parenchyma background in the MPVR images. For the part-solid nodule (PSN) view, the threshold was set at −350 HU to distinguish solid components from the ground-glass background. These threshold values were adopted from the established literature on quantitative CT analysis of lung nodules and correspond to the typical attenuation ranges that optimally separate the respective tissue densities (lung parenchyma vs. soft-tissue/vasculature; ground-glass vs. solid components) on TSCT [16] (see Supplementary Figure S1). The application of these thresholds in the present study was intended as a deliberate standardization measure, not as a re-validation within our specific cohort. This approach ensured inter-reader consistency and served the primary study objective of evaluating slab-thickness effects independently of threshold optimization. All parameters were locked and non-adjustable during image interpretation, thereby eliminating a potential source of operator-dependent bias and allowing radiologists to concentrate exclusively on diagnostic assessment. Furthermore, the MPVR post-processing tool was natively integrated into our institutional PACS workstation. This integration enabled radiologists to generate and review MPVR images directly within their standard diagnostic workflow, without the need for data export, external software, or platform switching, thus introducing no additional steps or time overhead. Blinded to pathological and clinical data, two senior radiologists (with more than 10 and 20 years of experience in chest CT interpretation, respectively) independently analyzed all of the TSCT and MPVR images. Then, observers were asked to record each patient’s clinical information, imaging characteristics, and reading time. Furthermore, another independent radiologist recorded the original diagnosis (based on TSCT imaging characteristics, and a consensus was reached by two radiologists before the final diagnosis). The following is an evaluation of the CT features of SSPN: lesion location (upper-right lobe, middle-right lobe, lower-right lobe, upper-left lobe, and lower-left lobe); solid surrounding heterogeneous area (homogeneous or heterogeneous); bundle sign; shape (round/oval or irregular); boundary (smooth or coarse/blurred); and whether there was pleural indentation, vascular bundle sign, margin, lobulation, spiculate, nodule diameter (the lesion is observed on the lung window and MPVR, and the maximum diameter is measured in coronal, axial, and sagittal planes, with the maximum value selected), solid component diameter (the lesion is observed on the lung window and MPVR, and the maximum solid component diameter is measured in coronal, axial, and sagittal planes, with the maximum value selected), and area (Manually outline the tumor’s boundaries, usually expressed in square millimeters (mm^2^)).

### 2.4. Pathologic Evaluation

All pathological specimens were obtained after surgical resection. The pathological diagnosis and classification were primarily based on the lung adenocarcinoma classification criteria published by the World Health Organization in 2021, which includes AAH, AIS, MIA, and IAC. All samples were fixed in 10% neutral formalin, and the cut sections were embedded in paraffin. They were then stained with hematoxylin and eosin, combined with Alcian blue-periodic acid-Schiff technique to assess the production of mucin in the cytoplasm. Hematoxylin–eosin-stained sections were randomly selected for review. If there was uncertainty in the histopathological classification under optical microscopy, immunohistochemical tests were performed for further confirmation. Two pathologists referenced the 2021 edition of the World Health Organization classification to identify the histological types and pathological grades of tumors in the lung [17]. The final histopathological classification for each case was established through consensus between the two pathologists, thereby ensuring diagnostic consistency for the study cohort.

### 2.5. Statistical Analysis

All statistical analyses were performed using SPSS (version 22.0, IBM). Between-group comparisons of quantitative variables were conducted using the independent samples *t*-test for normally distributed data or the Wilcoxon rank-sum test for non-normally distributed data. Categorical variables were compared using Pearson’s chi-square test or Fisher’s exact test, as appropriate.

Variable selection was performed using least absolute shrinkage and selection operator (LASSO) regression with 10-fold cross-validation to prevent overfitting and address multicollinearity among imaging features. The predictors selected by LASSO were then entered into a multivariable logistic regression model to derive the final parsimonious model. Model discrimination was evaluated by the area under the receiver AUC, with the optimal probability threshold determined by maximizing Youden’s J statistic. To correct for over-optimism, internal validation was conducted via bootstrap resampling with 2000 iterations. In each iteration, a model was developed on the bootstrap sample, and its performance was evaluated on both the bootstrap and the original sample. The average optimism (the difference between these two performances) was subtracted from the apparent performance of the full model to obtain the optimism-corrected AUC. Model calibration was assessed by plotting a calibration curve and calculating the calibration slope and intercept, along with their 95% confidence intervals, from the bootstrap distribution of the optimism-corrected predicted probabilities. For comparisons across the eight imaging models (one conventional and seven MPVR), pairwise differences in AUC were tested using DeLong’s test, with the resulting *p*-values adjusted for multiple comparisons using the Holm–Bonferroni method to control the family-wise error rate.

Missing data were handled using multiple imputations by chained equations (m = 20), and results were pooled following Rubin’s rules. The proportion of missing values was very low (< 2% for any variable), and these were isolated entries with no systematic pattern, justifying the use of imputation. Interobserver agreement was assessed using Cohen’s kappa for categorical variables and the intraclass correlation coefficient for continuous variables. Reading time data were summarized descriptively as mean ± standard deviation for each reader and imaging setting. The observed trends in reading time across different MPVR layer thicknesses and between lesion subtypes were compared descriptively to assess potential differences in interpretation efficiency.

## 3. Results

### 3.1. Characteristics of the Patients

Table 1 presents the characteristics of 601 patients in this study, including 197 in the non-IAC group (mean age, 59.0 years; range, 50.0–67.0 years) and 404 in the IAC group (mean age, 60.0 years; range, 53.0–67.0 years). In the non-IAC group, there were 79 female patients (40.10%) and 118 male patients (59.90%), while in the IAC group, there were 145 female patients (35.89%) and 259 male patients (64.11%). Analysis of nodule location revealed that nodules in both groups were predominantly located in the right upper lobe of the lung. Analysis of nodule density demonstrated a significant difference between the two groups (*p* < 0.001). In the non-IAC group, there were 101 PSNs (51.27%) and 96 solid nodules (SNs) (48.73%). In the IAC group, there were 124 PSNs (30.69%) and 280 SNs (69.31%). In addition, in the non-IAC group, there were 133 nodules with a size of ≤8 mm (67.5%) and 64 nodules with a size of 9–15 mm (32.5%). In the IAC group, there were 198 nodules with a size of ≤8 mm (49.0%) and 206 nodules with a size of 9–15 mm (51.0%). The total number of nodules with sizes of ≤ 8 mm and 9–15 mm across both groups was 331 (55.1%) and 270 (44.9%), respectively, with a statistical significance of *p* < 0.001.

### 3.2. Image Reading Time

The mean reading times for all imaging settings are summarized in Table 2. A consistent trend was observed wherein the mean reading time for the IAC group was higher than that for the non-IAC group across most MPVR thicknesses and for both readers. An observable trend indicated that reading time generally increased with MPVR layers thicker than 10 mm. Notably, the mean reading time for 10 mm MPVR images was among the shortest across all MPVR settings. Furthermore, the mean reading times for all MPVR layer thicknesses were lower than those for conventional lung window readings.

### 3.3. Inter-Observer Agreement

Interobserver agreement for key radiological features across the different MPVRs was good to excellent (Table 3). For categorical features, Cohen’s kappa ranged from 0.678 to 0.899 for boundary assessment, 0.791 to 0.873 for lobulation, and 0.712 to 0.863 for pleural indentation. For continuous measures, the intraclass correlation coefficient (ICC) was 0.893 (95% CI: 0.876–0.907) for solid component size and 0.891 (95% CI: 0.873–0.906) for total nodule diameter.

### 3.4. MPVR Image Feature Analysis by Different Layer Thickness

**Figure 2:**
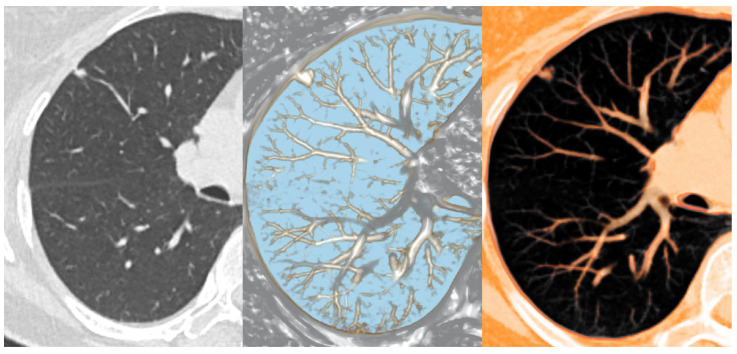
Thin-slab 1 mm axial CT view (**left**), nodule detection view of MPVR (**middle**), and PSN view of MPVR (**right**) from the CT data set of a 60-year-old patient with a pulmonary nodule predominantly composed of a solid component.

**Figure 3:**
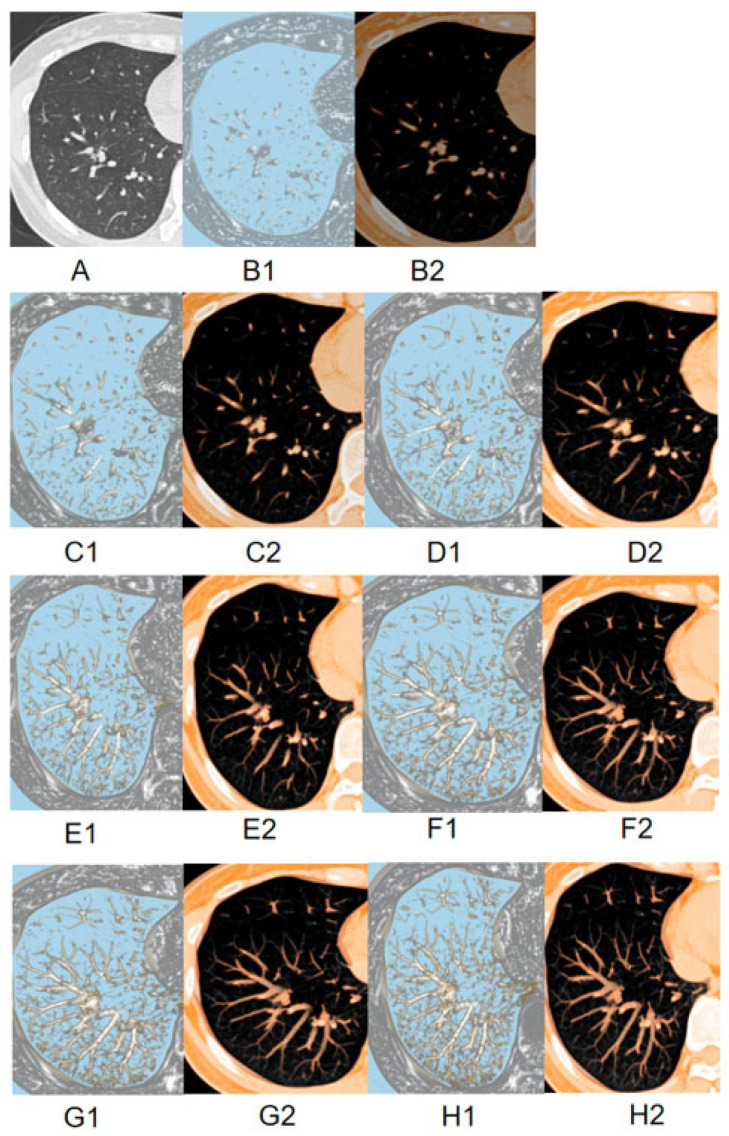
The image displays two-dimensional TSCT images (**A**); 2 mm MPVR model (**B**); 4 mm MPVR model (**C**); 6 mm MPVR model (**D**); 8 mm MPVR model (**E**); 10 mm MPVR model (**F**); 12 mm MPVR model (**G**); 14 mm MPVR model (**H**) from the CT dataset of a 57-year-old patient with a part-solid pulmonary nodule. For each thickness, the left panel (B1–H1) shows the nodule detection plane, and the right panel (B2–H2) shows the orthogonal plane for assessing the part-solid components.

We evaluated the imaging characteristics of solid component-dominant lung adenocarcinomas (≤ 15 mm) using lung window and MPVR images of varying layer thicknesses to predict the invasiveness of lung nodules. Univariate and multivariate logistic regression analyses were used for feature selection and model development (Supplementary Table S1). Additionally, performance metrics of the 10 mm MPVR model can be found in Table 4. The 10 mm MPVR model incorporated pleural indentation, boundary, shape, lobulation, spiculation, nodule diameter, solid component diameter, and area (Table 5 and Figure 2 and Figure 3). Multicollinearity among the predictors included in the final 10 mm MPVR model was assessed using variance inflation factors (VIFs). All VIF values were below 2.5, indicating no substantial multicollinearity (Supplementary Table S2). Thus, the final model incorporates a set of features that are both statistically stable and directly correspond to well-established radiological signs of invasiveness.

### 3.5. Assessment of Diagnostic Performance of Models

**Figure 4:**
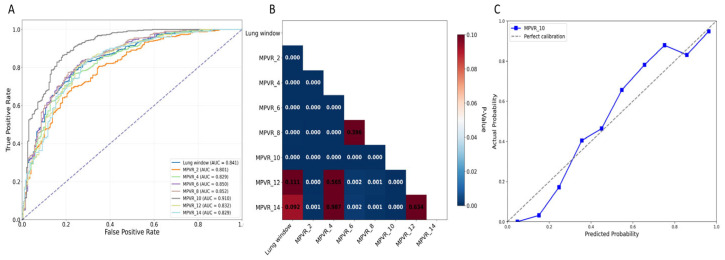
(**A**) ROC curves of different models for predicting invasive lung adenocarcinoma. (**B**) The heatmap for the results of the DeLong test between each model, with color intensity indicating the magnitude of difference. The performance of the 10 mm MPVR model was significantly different from the other ones. (**C**) The calibration curve of the 10 mm MPVR model. Abbreviations: AUC, area under the curve; MPVR, multi-planar volume reconstruction.

To compare the effects of different layer thicknesses of MPVRs on predicting the invasiveness of solid component-dominant adenocarcinomas (≤15 mm) and to identify the optimal thickness while evaluating its predictive accuracy and clinical utility, we created eight models. Among these, the 10 mm MPVR model demonstrated the highest diagnostic performance (AUC: 0.910, 95% CI: 0.905–0.914; sensitivity: 0.906; specificity: 0.753; accuracy: 0.856; PPV: 0.883; NPV: 0.796), with an optimal probability threshold of 0.583 (Figure 4 and Table 6). Model performance improved progressively as layer thickness increased from 2 mm to 10 mm, reflected by increases in sensitivity (0.870 to 0.906), specificity (0.519 to 0.753), and accuracy (0.755 to 0.856). Beyond the optimal 10 mm thickness, diagnostic performance declined, with the AUC, specificity, and accuracy decreasing at 12 mm and 14 mm thicknesses. The 10 mm MPVR model also significantly outperformed the lung window model (AUC: 0.841, 95% CI: 0.831–0.844).

Subgroup analyses confirmed the generalizability of the 10 mm MPVR model across key clinical strata using the predefined threshold of 0.583 (Table 6). It performed robustly across nodule types, with AUCs of 0.892 (95% CI: 0.865–0.919) for part-solid nodules and 0.901 (95% CI: 0.875–0.927) for solid nodules. When stratified by size, it maintained high accuracy for both ≤8 mm (AUC: 0.885, 95% CI: 0.855–0.915) and 9–15 mm (AUC: 0.908, 95% CI: 0.879–0.937) nodules, with marginally better performance in the latter (Table 7). Pairwise model comparisons further validated the superiority of the 10 mm MPVR model (Supplementary Table S3). After Holm–Bonferroni correction, its AUC was significantly greater than those of the lung window model and all MPVR models with thicknesses of 2 mm, 4 mm, 12 mm, and 14 mm (all *p* < 0.01). Although AUC point estimates were higher than those of the 6 mm and 8 mm models, these differences were not statistically significant after adjustment (*p* = 0.084 and *p* = 0.061, respectively).

### 3.6. Diagnostic Performance for Differentiating IAC from MIA

Given the clinical imperative to distinguish IAC from MIA—as this dictates divergent surgical strategies (lobectomy versus sublobar resection)—we specifically evaluated the 10 mm MPVR model for this binary classification task. The model demonstrated excellent discriminative ability, with an area under the curve (AUC) of 0.916 (95% CI: 0.889–0.943). Notably, it achieved a high positive predictive value (PPV) of 0.953 (95% CI: 0.927–0.973) for IAC. This indicates that when the model predicts IAC, it is correct in over 95% of cases, thereby providing strong support for confidently selecting patients for anatomic lobectomy (Table 8). The diagnostic performance of the 10 mm MPVR model in differentiating IAC from MIA was assessed.

## 4. Discussion

This study identified 10 mm as the optimal reconstruction layer thickness for MPVR to differentiate invasive from non-invasive/minimally invasive adenocarcinomas in nodules ≤15 mm. The 10 mm MPVR model provided significantly higher diagnostic accuracy than both conventional TSCT and other MPVR thicknesses. These findings confirm our primary hypothesis (H1) and the thickness-dependent performance posited in H2. Furthermore, the 10 mm protocol reduced interpretation time (H3) and achieved excellent inter-observer agreement (H4), demonstrating its efficiency and reproducibility for clinical use.

Diagnostic performance improved as MPVR layer thickness increased from 2 mm to 10 mm (AUC 0.801 to 0.910), highlighting a fundamental trade-off. The 10 mm thickness optimally compromises between spatial resolution and volume-averaging artifacts. Thinner slabs (2–8 mm) suffer from increased noise and partial-volume effects, obscuring fine features and limiting specificity. Excessively thick slabs (12–14 mm) cause blurring that smears critical discriminators like spiculation, reducing sensitivity. The 10 mm protocol balances these extremes; it supplies sufficient through-plane data to suppress noise and stabilize density measurements, improving feature quantifiability, yet retains enough z-axis resolution to clearly delineate invasive morphology. This balance accounts for the concurrent peak in AUC, sensitivity, and specificity at 10 mm.

Direct comparison with the next-best thickness (8 mm) reinforces this: the 10 mm model showed a significantly higher AUC (0.910 compared with 0.852, *p* < 0.01) and a clinically important gain in specificity (0.753 compared with 0.616). Therefore, increasing thickness from 8 mm to 10 mm enhanced overall discrimination while substantially reducing the false-positive rate—a key preoperative concern, as false-positive classification of invasiveness could prompt unnecessary lobectomy. For the pivotal clinical task of distinguishing IAC from MIA, which guides the choice between lobectomy and sublobar resection, the 10 mm MPVR model achieved an AUC of 0.916 and a PPV for IAC of 0.953. This high PPV indicates its potential to reliably select lesions for anatomic resection, thereby reducing the risk of undertreating invasive disease.

The superior diagnostic performance of the 10 mm MPVR model has direct clinical implications for preoperative planning. With a specificity of 0.753 and a high PPV of 0.883 for IAC, it reliably identifies nodules likely to require lobectomy. Conversely, an NPV of 0.796 allows confident exclusion of invasiveness in many nodules, facilitating the selection of candidates for sublobar resection or surveillance. This imaging-based stratification is essential to align surgical strategy with tumor biology, particularly given the markedly different prognoses between IAC and MIA/AIS.

These implications are especially relevant in East Asian populations, which have a high prevalence of indolent AIS and MIA among never smokers, a group poorly served by smoking-based risk models [2]. Here, our model’s capacity for accurate, single-time-point discrimination of invasiveness can help mitigate overtreatment without missing aggressive cancers. Its strong performance, specifically in differentiating IAC from MIA (AUC 0.916), directly supports its use in guiding the decisive choice between lobectomy and sublobar resection.

Current management of small solid-component nodules relies heavily on longitudinal surveillance, using interval growth as a proxy for invasive potential. Our study supports a complementary, cross-sectional approach. The 10 mm MPVR model provides immediate risk stratification by identifying high-risk morphology at a single time point, which is particularly useful for nodules exhibiting ambiguous or slow growth—scenarios where reliance on growth alone may delay intervention. Integrating this baseline morphological assessment with conventional growth monitoring may refine management, thus prompting earlier resection for morphologically aggressive nodules while justifying extended surveillance for those with indolent features.

The superior performance of 10 mm MPVR over conventional CT stems from the inherent advantages of volumetric, three-dimensional visualization. Standard axial review is limited to sequential slices, which can miss the full spatial configuration and subtle invasive details of small nodules. MPVR, by enabling multi-planar analysis and standardized segmentation, markedly improves the conspicuity of key discriminators like spiculation, lobulation, and pleural indentation. This enhanced visualization is critical for nodules ≤15 mm, where subtle signs of invasion are readily missed on 2D views, explaining the model’s gains in sensitivity and specificity.

Furthermore, the multivariate model revealed an inverse association between total nodule diameter and invasiveness (OR < 1), while larger solid component diameter strongly predicted invasiveness (OR > 1). This counterintuitive finding has a clear biological basis. In pulmonary adenocarcinomas, the solid component on CT directly correlates with the pathologically invasive focus, where cancer cells invade the stromal matrix—the primary driver of metastatic potential. Conversely, the total diameter often encompasses both this invasive focus and any associated non-invasive lepidic growth. Therefore, after adjusting for the size of the invasive solid component, a larger remaining total diameter predominantly reflects indolent lepidic growth, which is characteristic of pre-invasive or minimally invasive lesions. This underscores a critical clinical imperative: accurately isolating and quantifying the solid component is paramount for assessing invasiveness, as reliance on total nodule size alone can be misleading. MPVR, with its enhanced differentiation of tissue densities, is particularly well-suited to this task.

Our reading-time analysis further supports the practical utility of MPVR. Interpretation times for all MPVRs were consistently shorter than those for conventional lung window reading, confirming our hypothesis (H3) that MPVR can improve workflow efficiency. Notably, the 2 mm MPVR had the shortest reading time, which may reflect a rapid but potentially less definitive assessment due to higher image noise and reduced conspicuity of subtle features. In contrast, the 10 mm layer thickness achieved an optimal compromise; it maintained a significantly shorter reading time than both conventional CT and most other MPVR thicknesses, while delivering the highest diagnostic accuracy. Although the time saved per case may seem modest, when extrapolated to high-volume clinical practice, this efficiency gain can improve workflow throughput and potentially reduce interpreter fatigue without compromising diagnostic confidence. Excellent inter-observer agreement for key quantitative features on 10 mm MPVR images validates H4 and highlights the potential of this technique to standardize interpretation and reduce reader-dependent variability. These results, however, were obtained in a single-center setting with experienced radiologists under optimized conditions. While they demonstrate strong internal validity, multicenter studies involving readers of varying experience are necessary to confirm the generalizability and real-world utility of the 10 mm MPVR protocol. Pulmonary nodule management guidelines regard nodule size as the main malignant indicator, but this method is not effective in lesions with ≤15 mm because the aggressive characteristics of these lesions are often not obvious, while the 10 mm MPVR model bridges this gap by enhancing the detection of micro-infiltrating characteristics. In addition, prospective studies using large databases reported a significant increase in mortality due to delays in early-stage lung cancer treatment, with an average increase of 3.2% per week [18]. This suggests that delay has a very large impact on early-stage cancer, and MPVR may provide potential benefits for early-stage lung cancer prognosis. This accuracy is critical for personalized treatment, thereby optimizing oncology outcomes and quality of life.

Currently, researchers are focusing on using artificial intelligence-based detection tools, computer-assisted detection (CADe), to improve the efficiency of pulmonary nodules diagnosis and reduce misdiagnosis and missed diagnoses [19]. Although these emerging CADe systems show advantages and high efficiency in nodule detection, the integration of CADe with the daily workflow of radiologists remains challenging [20]. In clinical practice, radiologists typically first perform a quick review of 5 mm axial images and post-processed images to roughly locate candidate nodules, followed by a detailed evaluation of thinner slice images [21]. However, CADe tools are often not included in PACS software, requiring radiologists to switch to another software and repeat the image reading process, as CADe systems cannot present nodules in the preferred order of individual radiologists, which may increase the workload needed for accurate diagnosis [22,23,24]. In contrast, the MPVR technique used in this study is natively integrated into the PACS workstation, enabling “one-click” 3D reconstruction and evaluation without disrupting the diagnostic routine—a practical advantage corroborated by our time-efficiency analysis. Looking forward, the standardized, high-quality features extracted from the optimized 10 mm MPVR protocol could serve as ideal input for developing next-generation, PACS-embedded AI models. Such models could automatically quantify features that are challenging to assess visually, potentially further enhancing diagnostic performance and workflow efficiency.

This study has several limitations. First, the single-center retrospective design may introduce selection bias and limit the generalizability of the findings. Second, all CT data in this study were acquired using a single scanner model, which eliminates inter-scanner variability and ensures internal technical consistency for the developed MPVR protocol. However, this reliance on a single CT scanner model constrains the evaluation of protocol-dependent variability across different platforms; the optimized MPVR parameters may be influenced by scanner-specific technical characteristics and thus require adjustment and validation on other imaging systems. Third, although inter-reader agreement was high within our study, its reproducibility across readers with varying levels of experience and from other institutions requires further validation. Fourth, the model was developed and validated internally; external prospective validation is necessary to confirm its clinical robustness. Fifth, prospective studies with long-term follow-up are warranted to directly correlate the invasiveness predicted by our MPVR model with patient survival outcomes. Furthermore, while pathological diagnoses were made by consensus according to WHO standards, formal inter-pathologist agreement statistics were not calculated. Future studies could incorporate such metrics to further quantify diagnostic reproducibility. Finally, the inclusion criterion of only surgically resected and pathologically confirmed nodules may enrich the sample toward clinically suspicious lesions and may not fully represent the entire spectrum of incidentally detected small solid-dominant nodules.

## 5. Conclusions

This study demonstrates that MPVR with a 10 mm layer thickness provides optimal diagnostic performance for distinguishing invasive from non-invasive adenocarcinoma in solid-dominant pulmonary nodules ≤ 15 mm. The 10 mm-MPVR model protocol significantly outperformed conventional TSCT, offering a superior balance of sensitivity and specificity. Its integration into the clinical workflow reduced image interpretation time while maintaining excellent inter-observer agreement. These findings validate the 10 mm MPVR as an efficient and reproducible non-invasive tool, with the potential to inform more precise preoperative planning for early-stage lung adenocarcinoma.

## Figures and Tables

**Table 1:** Clinical characteristics of patients and nodules.

Characteristics		Invasive Subtype of Lung Adenocarcinoma		Total (n = 601)	*p*
		Non-IAC (n = 197)	IAC (n = 404)		
Age (years)		59.00 (50.00, 67.00)	60.00 (53.00, 67.00)		0.253
Gender	Female	79 (40.10)	145 (35.89)	224 (37.27)	0.316
	Male	118 (59.90)	259 (64.11)	377 (62.73)	
Nodule location	Upper-right lobe	76 (38.58)	140 (34.65)	216 (35.94)	0.650
	Middle-right lobe	17 (8.63)	33 (8.17)	50 (8.32)	
	Lower-right lobe	28 (14.21)	53 (13.12)	81 (13.48)	
	Upper-left lobe	59 (29.95)	147 (36.39)	206 (34.28)	
	Lower-left lobe	17 (8.63)	31 (7.67)	48 (7.99)	
Density	PSN	101 (51.27)	124 (30.69)	225 (37.43)	<0.001
	SN	96 (48.73)	280 (69.31)	376 (62.56)	
Size strata	≤8 mm	133 (67.50)	198 (49.00)	331 (55.07)	<0.001
	9–15 mm	64 (32.50)	206 (51.00)	270 (44.93)	

PSN, partially solid nodule; SN, solid nodule; IAC, invasive adenocarcinoma.

**Table 2:** Reading time for non-IAC and IAC groups across different MPVR settings.

Reading Time (s)	Invasive Subtype of Lung Adenocarcinoma	
	Non-IAC (n = 197)	IAC (n = 404)
Reader1_lung-window Reading time (s)	172 ± 15	173 ± 34
Reader2_lung-window Reading time (s)	156 ± 34	167 ± 38
Reader1_MPVR_2Reading time (s)	82 ± 13	83 ± 18
Reader2_MPVR_2Reading time (s)	70± 15	73 ± 20
Reader1_MPVR_4Reading time (s)	153 ± 28	174± 27
Reader2_MPVR_4Reading time (s)	157± 22	168± 10
Reader1_MPVR_6Reading time (s)	156 ± 14	187 ± 29
Reader2_MPVR_6Reading time (s)	158 ± 14	174 ± 19
Reader1_MPVR_8Reading time (s)	150 ± 24	184 ± 25
Reader2_MPVR_8Reading time (s)	142 ± 25	164 ± 20
Reader1_MPVR_10Reading time (s)	105± 28	115± 27
Reader2_MPVR_10Reading time (s)	113± 14	116 ± 20
Reader1_MPVR_12Reading time (s)	117 ± 25	130 ± 20
Reader2_MPVR_12Reading time (s)	124 ± 25	134 ± 20
Reader1_MPVR_14Reading time (s)	123 ± 27	151± 27
Reader2_MPVR_14Reading time (s)	137± 22	164± 15

Data are presented as mean ± standard deviation (s). MPVR, multi-planar volume reconstruction.

**Table 3:** Interobserver agreement.

	Boundary	Lobulation	Pleural Indentation	MPVR Solid Component Size	Nodule Diameter
Interobserver Agreement (95% CI)	κ = 0.678–0.899	κ = 0.791–0.873	κ = 0.712–0.863	ICC = 0.893 (95% CI: 0.876–0.907)	ICC = 0.891 (95% CI: 0.873–0.906)

Agreement is reported as κ for categorical variables and ICC for continuous variables, with 95% CIs. MPVR, multi-planar volume reconstruction.

**Table 4:** Performance metrics of the 10 mm MPVR model.

Predictor Variable	β	SE	OR (95% CI)	*p*-Value
Pleural indentation	0.816	0.278	2.263 (1.324–3.866)	0.003
Shape	−0.547	0.311	0.579 (0.311~1.075)	0.084
Boundary	−1.062	0.298	0.346 (0.194–0.616)	<0.001
Lobulation	2.481	0.423	11.949 (5.897–24.211)	<0.001
Spiculation	0.902	0.331	2.465 (1.277–4.757)	0.007
Nodule diameter	−0.821	0.148	0.440 (0.330–0.588)	<0.001
Solid component diameter	0.729	0.107	2.074 (1.679–2.564)	<0.001
Area	0.029	0.012	1.029 (1.006–1.052)	0.015

All features included were selected through LASSO regression and demonstrated significant differences. Abbreviations: CI, confidence interval; OR, Odds Ratio; SE, Standard Error.

**Table 5:** Univariate and multivariate logistic regression analyses of 10 mm MPVR model imaging characteristics for predicting invasive adenocarcinoma.

Characteristic	Univariate Analysis		Multivariate Analysis	
	OR Value (95% CI)	*p*-Value	OR Value (95% CI)	*p*-Value
Age (years)	0.989 (0.975~1.003)	0.124	—	—
Gender	1.196 (0.843~1.697)	0.317	—	—
Location	1.063 (0.945~1.196)	0.308	—	—
Solid surrounding heterogeneous area	1.611 (1.134~1.877)	0.66	—	—
Pleural indentation	2.116 (2.572~6.037)	<0.001	2.263 (1.324~3.866)	0.003
Vascular bundle sign	1.134 (0.742~1.735)	0.561	—	—
Shape	0.557 (0.356~0.871)	0.010	0.579 (0.311~1.075)	0.084
Boundary	0.603 (0.408~0.893)	0.012	0.346 (0.194~0.616)	<0.001
Margin	0.411 (0.290~0.583)	<0.001	0.661 (0.391~1.119)	0.123
Lobulation	16.361 (8.840~30.279)	<0.001	11.949 (5.897~ 24.211)	<0.001
Spiculate	5.245 (3.134~8.777)	<0.001	2.465 (1.277~4.757)	0.007
Nodule diameter	1.472 (1.341~1.616)	<0.001	0.440 (0.330~0.588)	<0.001
Solid component diameter	1.654 (1.510~1.811)	<0.001	2.074 (1.679~2.564)	<0.001
Area	1.041 (1.032~1.050)	<0.001	1.029 (1.006~1.052)	0.015

CI, confidence interval; MPVR, multiplanar volume rendering; —, not applicable; OR, odds ratio.

**Table 6:** Diagnostic performance of different imaging models in predicting invasive adenocarcinomas.

Model	Sensitivity	Specificity	Accuracy	PPV	NPV	AUC	Optimal Threshold
Lung window	0.875 (0.844, 0.908)	0.593 (0.515, 0.656)	0.783 (0.768, 0.795)	0.816 (0.793, 0.835)	0.699 (0.661, 0.743)	0.841 (0.831, 0.844)	0.628
2 mm MPVR Model	0.870 (0.809, 0.918)	0.519 (0.429, 0.597)	0.755 (0.732, 0.774)	0.789 (0.766, 0.809)	0.662 (0.597, 0.726)	0.801 (0.786, 0.804)	0.686
4 mm MPVR Model	0.876 (0.840, 0.911)	0.569 (0.495, 0.633)	0.776 (0.762, 0.790)	0.808 (0.786, 0.827)	0.691 (0.650, 0.738)	0.829 (0.818, 0.832)	0.653
6 mm MPVR Model	0.881 (0.849, 0.913)	0.613 (0.546, 0.668)	0.794 (0.780, 0.806)	0.825 (0.805, 0.843)	0.716 (0.679, 0.760)	0.850 (0.842, 0.854)	0.596
8 mm MPVR Model	0.881 (0.849, 0.916)	0.616 (0.551, 0.673)	0.795 (0.783, 0.805)	0.826 (0.807, 0.843)	0.717 (0.680, 0.762)	0.852 (0.843, 0.855)	0.647
10 mm MPVR Model	0.906 (0.877, 0.931)	0.753 (0.704, 0.793)	0.856 (0.843, 0.868)	0.883 (0.866, 0.898)	0.796 (0.755, 0.840)	0.910 (0.905, 0.914)	0.583
12 mm MPVR Model	0.884 (0.849, 0.913)	0.598 (0.520, 0.671)	0.791 (0.775, 0.807)	0.820 (0.795, 0.843)	0.716 (0.675, 0.754)	0.832 (0.819, 0.833)	0.626
14 mm MPVR Model	0.883 (0.848, 0.913)	0.610 (0.541, 0.668)	0.794 (0.777, 0.808)	0.824 (0.802, 0.841)	0.718 (0.673, 0.755)	0.829 (0.819, 0.833)	0.571

Values are expressed as a number (95% CI). PPV, positive predictive value; NPV, negative predictive value; AUC, area under the curve; MPVR, multi-planar volume reconstruction; CI, confidence interval.

**Table 7:** Stratified diagnostic performance of the 10 mm MPVR model.

Subgroup	n	AUC	Sensitivity	Specificity
By Density				
PSN	225	0.892 (0.865–0.919)	0.887 (0.825–0.937)	0.742 (0.662–0.813)
SN	376	0.901 (0.875–0.927)	0.914 (0.876–0.947)	0.760 (0.683–0.829)
By Size				
≤8 mm	331	0.885 (0.855–0.915)	0.894 (0.847–0.934)	0.729 (0.654–0.798)
9–15 mm	270	0.908 (0.879–0.937)	0.917 (0.861–0.958)	0.780 (0.707–0.844)

Values are presented as the point estimate (95% confidence interval).

**Table 8:** Performance of the 10 mm MPVR model in discriminating between invasive and minimally invasive adenocarcinomas.

Model Comparison	n (IAC/MIA)	AUC	Sensitivity	Specificity	PPV	NPV	Accuracy
IAC vs. MIA	404/80	0.916 (0.889–0.943)	0.906 (0.877–0.931)	0.775 (0.675–0.856)	0.953 (0.927–0.973)	0.620 (0.520–0.713)	0.884 (0.852–0.912)

IAC, invasive adenocarcinoma; MIA, minimally invasive adenocarcinoma.

## Data Availability

The datasets generated or analyzed during the study are available from the corresponding author upon reasonable request.

## Supplementary Materials

The following supporting information can be downloaded at: https://www.mdpi.com/article/10.3390/diagnostics16020220/s1, Figure S1: Opacity and pseudo-color curve settings of MPVR; Table S1: Univariate and Multivariate Logistic Regression Analysis of Imaging Features in Lung Window and MPVR with Different Slice Thicknesses. Table S2: VIF for predictors in the final 10 mm MPVR model. Table S3: Pairwise DeLong *p*-values for model AUCs; Supplementary File S1. TRIPOD Checklist: Prediction Model Development and Validation.

## References

[1] Rebecca L.S., Deepa N., Ahmedin J. (2013). Cancer statistics, 2013. CA Cancer J. Clin..

[2] Wu F.Z., Huang Y.L., Wu C.C., Tang E.K., Chen C.S., Mar G.Y., Yen Y., Wu M.T. (2016). Assessment of Selection Criteria for Low-Dose Lung Screening CT Among Asian Ethnic Groups in Taiwan: From Mass Screening to Specific Risk-Based Screening for Non-Smoker Lung Cancer. Clin. Lung Cancer.

[3] Travis W.D., Brambilla E., Noguchi M., Nicholson A.G., Geisinger K.R., Yatabe Y., Beer D.G., Powell C.A., Riely G.J., Van Schil P.E. (2011). International Association for the Study of Lung Cancer/American Thoracic Society/European Respiratory Society International Multidisciplinary Classification of Lung Adenocarcinoma. J. Thorac. Oncol..

[4] Martin M.D., Henry T.S., Berry M.F., Johnson G.B., Kelly A.M., Ko J.P., Kuzniewski C.T., Lee E., Maldonado F., Morris M.F. (2023). ACR Appropriateness Criteria^®^ Incidentally Detected Indeterminate Pulmonary Nodule. J. Am. Coll. Radiol..

[5] McWilliams A., Tammemagi M.C., Mayo J.R., Roberts H., Liu G., Soghrati K., Yasufuku K., Martel S., Laberge F., Gingras M. (2013). Probability of Cancer in Pulmonary Nodules Detected on First Screening CT. N. Engl. J. Med..

[6] Yip R., Henschke C.I., Yankelevitz D.F., Smith J.P. (2014). CT Screening for Lung Cancer: Alternative Definitions of Positive Test Result Based on the National Lung Screening Trial and International Early Lung Cancer Action Program Databases. Radiology.

[7] She Y.L. (2016). 101PD: Preoperative nomogram for identifying invasive pulmonary adenocarcinoma in patients with pure ground-glass nodule: A multi-institutional study. J. Thorac. Oncol..

[8] Song X., Zhao Q., Zhang H., Xue W., Xin Z., Xie J., Zhang X. (2022). Development and Validation of a Preoperative CT-Based Nomogram to Differentiate Invasive from Non-Invasive Pulmonary Adenocarcinoma in Solitary Pulmonary Nodules. Cancer Manag. Res..

[9] Meier-Schroers M., Homsi R., Skowasch D., Buermann J., Zipfel M., Schild H.H., Thomas D. (2017). Lung cancer screening with MRI: Results of the first screening round. J. Cancer Res. Clin. Oncol..

[10] Lee S.M., Park C.M., Goo J.M., Lee H.J., Wi J.Y., Kang C.H. (2013). Invasive Pulmonary Adenocarcinomas versus Preinvasive Lesions Appearing as Ground-Glass Nodules: Differentiation by Using CT Features. Radiology.

[11] Park S., Park G., Lee S.M., Kim W., Park H., Jung K., Seo J.B. (2021). Deep learning–based differentiation of invasive adenocarcinomas from preinvasive or minimally invasive lesions among pulmonary subsolid nodules. Eur. Radiol..

[12] Duran A.H., Duran M.N., Masood I., Maciolek L.M., Hussain H. (2019). The Additional Diagnostic Value of the Three-dimensional Volume Rendering Imaging in Routine Radiology Practice. Cureus.

[13] Zachary A., Erica C.K. (2023). Three-dimensional Visualization in Diagnostic Imaging: A Renaissance in Radiology. Radiology.

[14] Xiong J.W., Tao Y., Li W.J. (2024). Analyzing the clinical significance of MPVR technology in diagnosing invasive pulmonary ground-glass nodules. Chin. J. Lung Dis. (Electron. Ed.).

[15] Li W.J., Chu Z.G., Li D., Jing W.W., Shi Q.L., Lv F.J. (2024). Accuracy of solid portion size measured on multiplanar volume rendering images for assessing invasiveness in lung adenocarcinoma manifesting as subsolid nodules. Quant. Imaging Med. Surg..

[16] Li Y., Lu L., Xiao M., Dercle L., Huang Y., Zhang Z., Schwartz L.H., Li D., Zhao B. (2018). CT Slice Thickness and Convolution Kernel Affect Performance of a Radiomic Model for Predicting EGFR Status in Non-Small Cell Lung Cancer: A Preliminary Study. Sci. Rep..

[17] Nicholson A.G., Tsao M.S., Beasley M.B., Borczuk A.C., Brambilla E., Cooper W.A., Dacic S., Jain D., Kerr K.M., Lantuejoul S. (2022). The 2021 WHO Classification of Lung Tumors: Impact of Advances Since 2015. J. Thorac. Oncol..

[18] Yankelevitz D.F., Yip R., Henschke C.I. (2023). Impact of Duration of Diagnostic Workup on Prognosis for Early Lung Cancer. J. Thorac. Oncol..

[19] Bi W.L., Hosny A., Schabath M.B., Giger M.L., Birkbak N.J., Mehrtash A., Allison T., Arnaout O., Abbosh C., Dunn I.F. (2019). Artificial intelligence in cancer imaging: Clinical challenges and applications. CA Cancer J. Clin..

[20] Aggarwal R., Sounderajah V., Martin G., Ting D.S., Karthikesalingam A., King D., Ashrafian H., Darzi A. (2021). Diagnostic accuracy of deep learning in medical imaging: A systematic review and meta-analysis. Npj Digit. Med..

[21] Kilburn-Toppin F., Arthurs O.J., Tasker A.D., Set P.A. (2013). Detection of pulmonary nodules at paediatric CT: Maximum intensity projections and axial source images are complementary. Pediatr. Radiol..

[22] Gil J., Choi H., Paeng J.C., Cheon G.J., Kang K.W. (2023). Deep Learning-Based Feature Extraction from Whole-Body PET/CT Employing Maximum Intensity Projection Images: Preliminary Results of Lung Cancer Data. Nucl. Med. Mol. Imaging.

[23] Wu Y., Qi Q., Qi S., Yang L., Wang H., Yu H., Li J., Wang G., Zhang P., Liang Z. (2023). Classification of COVID-19 from community-acquired pneumonia: Boosting the performance with capsule network and maximum intensity projection image of CT scans. Comput. Biol. Med..

[24] Zheng S., Guo J., Cui X., Veldhuis R.N., Oudkerk M., Van Ooijen P.M. (2020). Automatic Pulmonary Nodule Detection in CT Scans Using Convolutional Neural Networks Based on Maximum Intensity Projection. IEEE Trans. Med. Imaging.

