# Supplementary material for: Optimizing Layer Thickness in Multi-Planar Volume Reconstruction for Distinguishing Invasive Adenocarcinoma from Non-Invasive and Minimally Invasive Lesions in Pulmonary Nodules (≤15 mm): A Comparative Study with Conventional Lung Window Settings
Source: Diagnostics (Basel). 2026 Jan 9;16(2):220. doi: 10.3390/diagnostics16020220 (PMC12840378; doi:10.3390/diagnostics16020220)
Supplement: Supplementary file 1 [file diagnostics-16-00220-s001.zip › Supplemenary file S1.pdf]

TRIPOD Checklist: Prediction Model Development and Validation

Section	Item		Checklist description	Reported on Page	Reported on Section/Paragraph
**Title and abstract**					
Title	1	D;V	Identify the study as developing and/or validating a multivariable prediction model, the target population, and the outcome to be predicted.	Page1	Paragraph2-5
Abstract	2	D;V	Provide a summary of objectives, study design, setting, participants, sample size, predictors, outcome, statistical analysis, results, and conclusions.	Page1-2	Paragraph28-56
**Introduction**					
Background and objectives	3a	D;V	Explain the medical context (including whether diagnostic or prognostic) and rationale for developing or validating the multivariable prediction model, including references to	Page2	Paragraph57-90
	3b	D;V	Specify the objectives, including whether the study describes the development or validation of the model or both.	Page2-3	Paragraph91-95
**Methods**					
Source of data	4a	D;V	Describe the study design or source of data (e.g., randomized trial, cohort, or registry data), separately for the development and validation data sets, ifapplicable.	Page3	Paragraph98-102
	4b	D;V	Specify the key study dates, including start of accrual; end of accrual; and, if applicable, end of follow-up.	Page3	Paragraph98-102
Participants	5a	D;V	Specify key elements of the study setting (e.g., primary care, secondary care, general population) including number and location of centres.	Page3	Paragraph98-102
	5b	D;V	Describe eligibility criteria for participants.	Page3	Paragraph102-110
	5c	D;V	Give details of treatments received, if relevant.	N/A	N/A

Outcome	6a	D;V	Clearly define the outcome that is predicted by the prediction model, including how and when assessed.	Page1	**Paragraph29-31**
	6b	D;V	Report any actions to blind assessment of the outcome to be predicted.	Page4	**Paragraph138-140**
Predictors	7a	D;V	Clearly define all predictors used in developing or validating the multivariable prediction model, including how and when they were measured.	Page4	**Paragraph141-151**
	7b	D;V	Report any actions to blind assessment of predictors for the outcome and other	Page4	**Paragraph134-136**
Sample size	8	D;V	Explain how the study size was arrived at.	Page3	**Paragraph109-110**

Missing data	9	D;V	Describe how missing data were handled (e.g., complete-case analysis, single imputation, multiple imputation) with details of any imputation method.	Page5	**Paragraph187-188**
Statistical analysis methods	10a	D	Describe how predictors were handled in the analyses.	Page5;7	**Paragraph175-177;241-243**
	10b	D	Specify type of model, all model-building procedures (including any predictor selection), and method for internal validation.	Page5	**Paragraph180-182**
	10c	V	For validation, describe how the predictions were calculated.	Page5	**Paragraph180-182**
	10d	D;V	Specify all measures used to assess model performance and, if relevant, to compare multiple models.	Page5	**Paragraph178-180;182-186**
	10e	V	Describe any model updating (e.g., recalibration) arising from the validation, if done.	N/A	N/A
Risk groups	11	D;V	Provide details on how risk groups were created, if done.	N/A	N/A
Development vs. validation	12	V	For validation, identify any differences from the development data in setting, eligibility criteria, outcome, and predictors.	Page3	**Paragraph109-110**
**Results**					
Participants	13a	D;V	Describe the flow of participants through the study, including the number of participants with and without the outcome and, if applicable, a summary of the follow-up time. A diagram may be helpful.	Page3	**Paragraph110-111**
	13b	D;V	Describe the characteristics of the participants (basic demographics, clinical features, available predictors), including the number of participants with missing data for predictors and outcome.	Page5	**Paragraph193-209**

	13c	V	For validation, show a comparison with the development data of the distribution of important variables (demographics, predictors and outcome).	Page5	**Paragraph193-209**
Model developme	14a	D	Specify the number of participants and outcome events in each analysis.	Page5	**Paragraph193-214**
	14b	D	If done, report the unadjusted association between each candidate predictor and outcome.	Page8-9	**Paragraph252-254**
Model specificatio n	15a	D	Present the full prediction model to allow predictions for individuals (i.e., all regression coefficients, and model intercept or baseline survival at a given time point).	Page8	**Paragraph248-251**
	15b	D	Explain how to the use the prediction model.	Page10	**Paragraph269-293**
Model performan	16	D;V	Report performance measures (with CIs) for the prediction model.	Page11	**Paragraph301-303**
Model-updating	17	V	If done, report the results from any model updating (i.e., model specification, model	N/A	N/A
**Discussion**					
Limitations	18	D;V	Discuss any limitations of the study (such as nonrepresentative sample, few events per predictor, missing data).	Page14	**Paragraph411-420**

Interpretation	19a	V	For validation, discuss the results with reference to performance in the development data, and any other validation data.	Page10	**Paragraph270-280**
	19b	D;V	Give an overall interpretation of the results, considering objectives, limitations, and results from similar studies, and other relevant evidence.	Page12	**Paragraph311-322**
Implications	20	D;V	Discuss the potential clinical use of the model and implications for future research.	Page12-13	**Paragraph323-338**
**Other information**					
Supplementary	21	D;V	Provide information about the availability of supplementary resources, such as study protocol, Web calculator, and data sets.	Page15	**Paragraph430-433**
Funding	22	D;V	Give the source of funding and the role of the funders for the present study.	Page15	**Paragraph440-444**

* Items relevant only to the development of a prediction model are denoted by D, items relating solely to a validation of a prediction model are denoted by V, and items relating to both are denoted D;V. We recommend using the TRIPOD Checklist in conjunction with the TRIPOD Explanation and Elaboration document.

Please leave this space alone as it will be supplemented by the editorial office when needed.
