# Supplementary material for: Optimizing Layer Thickness in Multi-Planar Volume Reconstruction for Distinguishing Invasive Adenocarcinoma from Non-Invasive and Minimally Invasive Lesions in Pulmonary Nodules (≤15 mm): A Comparative Study with Conventional Lung Window Settings
Source: Diagnostics (Basel). 2026 Jan 9;16(2):220. doi: 10.3390/diagnostics16020220 (PMC12840378; doi:10.3390/diagnostics16020220)
Supplement: Supplementary file 1 [file diagnostics-16-00220-s001.zip › Supplementary Figures and Tables.pdf]

Supplementary Figure S1. Opacity and pseudo-color curve settings of MPVR.

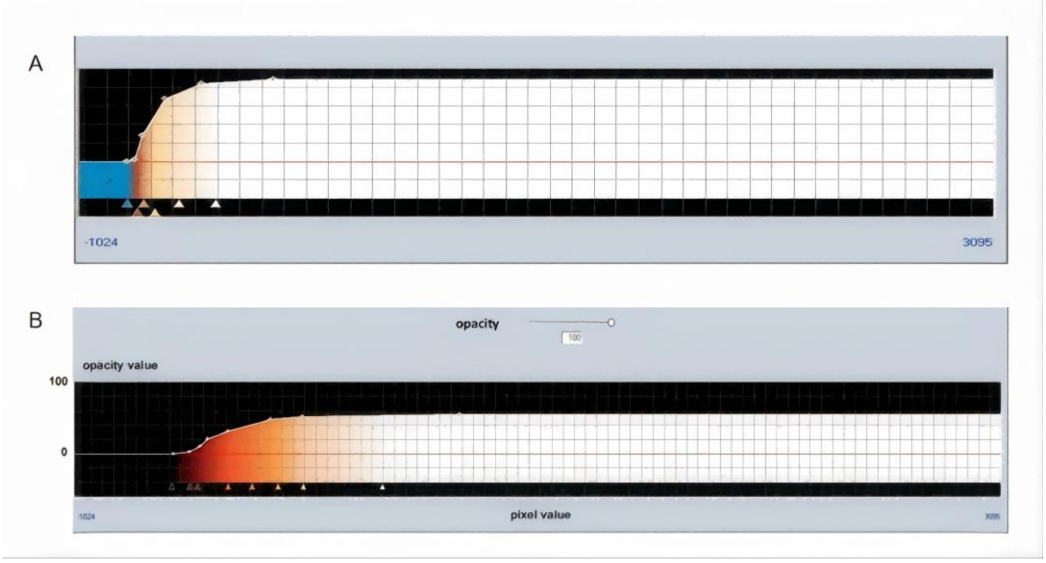

Nodule detection view (**A**) ,which displays the opacity curve (blue line) and pseudo-color map, featuring a primary threshold (arrow) to differentiate higher-density structures and PSN view (**B**), which employs adjusted transfer curves with a distinct threshold (arrow) to differentiate internal components.

Supplementary Table S1. Univariate and Multivariate Logistic Regression Analysis of Imaging Features in Lung Window and MPVR with Different Slice Thicknesses.

Characteristic	Lung Window				MPVR_2				MPVR_4				MPVR_6			
	Univariate Analysis		Multivariate Analysis		Univariate Analysis		Multivariate Analysis		Univariate Analysis		Multivariate Analysis		Univariate Analysis		Multivariate Analysis	
	OR Value (95%CI)	*p* Value	OR Value (95%CI)	*p* Value	OR Value (95%CI)	*p* Value	OR Value (95%CI)	*p* Value	OR Value (95%CI)	*p* Value	OR Value (95%CI)	*p* Value	OR Value (95%CI)	*p* Value	OR Value (95%CI)	*p* Value
Age (years)	0.989 (0.975 ~ 1.003)	0.124	——	——	0.989 (0.975 ~ 1.003)	0.124	——	——	0.989 (0.975 ~ 1.003)	0.124	——	——	0.989 (0.975 ~ 1.003)	0.124	——	——
Gender	1.196 (0.843 ~ 1.697)	0.317	——	——	1.196 (0.843 ~ 1.697)	0.317	——	——	1.196 (0.843 ~ 1.697)	0.317	——	——	1.196 (0.843 ~ 1.697)	0.317	——	——
Location	1.063 (0.945 ~ 1.196)	0.308	——	——	1.063 (0.945 ~ 1.196)	0.308	——	——	1.063 (0.945 ~ 1.196)	0.308	——	——	1.063 (0.945 ~ 1.196)	0.308	——	——
Solid surrounding heterogeneous area	1.513(1.026 ~ 2.232)	0.037	2.516(1.437 ~ 4.405)	≤ 0.001	0.666(0.455 ~ 0.975)	0.037	0.941(0.570 ~ 1.552)	0.812	1.161(0.762 ~ 1.770)	0.487	——	——	1.644(1.014 ~ 1.770)	0.567	——	——
Pleural indentation	3.181(2.158 ~ 4.688)	≤ 0.001	2.754(1.678 ~ 4.518)	≤ 0.001	9.903(4.513 ~ 21.731)	≤ 0.001	2.326(1.225 ~ 4.417)	0.01	5.856(3.269 ~ 10.491)	≤ 0.001	3.607(1.886 ~ 6.899)	≤ 0.001	9.901(4.511 ~ 21.722)	≤ 0.001	2.121(1.234 ~ 3.647)	0.007
Vascular bundle sign	1.407(0.951 ~ 2.081)	0.087	——	——	2.138(1.442 ~ 3.168)	≤ 0.001	1.499(0.965 ~ 2.330)	0.072	1.647(1.158 ~ 2.342)	0.005	1.126(0.812 ~ 1.746)	0.36	1.455(1.023 ~ 2.069)	0.037	0.83(0.535 ~ 1.287)	0.405
Shape	0.557(0.356 ~ 0.871)	0.01	——	——	0.533(0.340 ~ 0.836)	0.006	0.625(0.368 ~ 1.061)	0.082	1.834(1.171 ~ 2.872)	0.008	1.871(1.082 ~ 3.238)	0.025	1.834(1.174 ~ 2.877)	0.008	0.485(0.273 ~ 0.862)	0.014
Boundary	0.701(0.478 ~ 1.027)	0.068	——	——	0.635(0.429 ~ 0.941)	0.024	0.704(0.434 ~ 1.144)	0.156	0.614(0.415 ~ 0.909)	0.015	0.464(0.278 ~ 0.775)	0.003	0.593(0.399 ~ 0.879)	0.009	0.408(0.238 ~ 0.699)	≤ 0.001
Margin	0.422(0.312 ~ 0.627)	≤ 0.001	0.629(0.385 ~ 1.027)	0.064	0.412(0.289 ~ 0.588)	≤ 0.001	0.72(0.458 ~ 1.131)	0.154	0.412(0.290 ~ 0.586)	≤ 0.001	0.708(0.446 ~ 1.124)	0.143	0.413(0.290 ~ 0.587)	≤ 0.001	0.799(0.493 ~ 1.295)	0.362
Lobulation	10.800(6.330 ~ 18.425)	≤ 0.001	8.461(4.626 ~ 15.476)	≤ 0.001	4.577(1.603 ~ 13.065)	0.004	1.162(0.434 ~ 3.108)	0.765	5.897(2.088 ~ 16.658)	0.001	3(0.985 ~ 9.135)	0.053	13.140(5.260 ~ 32.828)	≤ 0.001	7.993(2.996 ~ 21.327)	≤ 0.001
Spiculate	8.023(4.218 ~ 15.260)	≤ 0.001	3.491(1.659 ~ 7.345)	≤ 0.001	6.517(0.846 ~ 50.176)	0.027	1.116(0.237 ~ 5.264)	0.89	11.832(1.586 ~ 88.264)	0.016	9.676(1.021 ~ 91.740)	0.048	20.788(5.043 ~ 85.691)	≤ 0.001	10.714(2.218 ~ 51.765)	0.003
Nodule diameter	1.386(1.258 ~ 1.527)	≤ 0.001	0.574(0.466 ~ 0.707)	≤ 0.001	1.216(1.136 ~ 1.301)	≤ 0.001	0.624(0.509 ~ 0.764)	≤ 0.001	1.218(1.139 ~ 1.304)	≤ 0.001	0.497(0.404 ~ 0.611)	≤ 0.001	1.339 (1.226~1.473)	≤ 0.001	0.531(0.405 ~ 0.697)	≤ 0.001
Solid component diameter	1.663(1.508 ~ 1.833)	≤ 0.001	2.108(1.744 ~ 2.549)	≤ 0.001	1.362(1.239 ~ 1.414)	≤ 0.001	1.627(1.365 ~ 1.939)	≤ 0.001	1.370(1.275 ~ 1.471)	≤ 0.001	1.768(1.487 ~ 2.102)	≤ 0.001	1.882(1.575~2.144)	≤ 0.001	1.949(1.626 ~ 2.337)	≤ 0.001
Area	1.035 (1.026-1.044)	<0.001	1.020 (1.008-1.032)	0.001	1.028 (1.019-1.037)	<0.001	1.015 (1.003-1.027)	0.015	1.030 (1.021-1.039)	<0.001	1.018 (1.006-1.030)	0.005	1.033 (1.024-1.042)	<0.001	1.022 (1.010-1.034)	<0.001

Characteristic	MPVR_8				MPVR_10				MPVR_12				MPVR_14			
	Univariate Analysis		Multivariate Analysis		Univariate Analysis		Multivariate Analysis		Univariate Analysis		Multivariate Analysis		Univariate Analysis		Multivariate Analysis	
	OR Value (95%CI)	*p* Value	OR Value (95%CI)	*p* Value	OR Value (95%CI)	*p* Value	OR Value (95%CI)	*p* Value	OR Value (95%CI)	*p* Value	OR Value (95%CI)	*p* Value	OR Value (95%CI)	*p* Value	OR Value (95%CI)	*p* Value
Age (years)	0.989 (0.975 ~ 1.003)	0.124	——	——	0.989 (0.975 ~ 1.003)	0.124	——	——	0.989 (0.975 ~ 1.003)	0.124	——	——	0.989 (0.975 ~ 1.003)	0.124	——	——
Gender	1.196 (0.843 ~ 1.697)	0.317	——	——	1.196 (0.843 ~ 1.697)	0.317	——	——	1.196 (0.843 ~ 1.697)	0.317	——	——	1.196 (0.843 ~ 1.697)	0.317	——	——
Location	1.063 (0.945 ~ 1.196)	0.308	——	——	1.063 (0.945 ~ 1.196)	0.308	——	——	1.063 (0.945 ~ 1.196)	0.308	——	——	1.063 (0.945 ~ 1.196)	0.308	——	——
Solid surrounding heterogeneous area	0.507(0.351 ~ 0.732)	<0.001	0.631(0.383 ~ 1.042)	0.072	1.611(1.134 ~ 1.877)	0.66	——	——	1.213(0.997 ~ 1.820)	0.466	——	——	1.233(1.011 ~ 1.918)	0.548	——	——
Pleural indentation	9.803(4.413 ~ 21.631)	<0.001	1.879(1.102 ~ 3.206)	0.021	2.116(2.572 ~ 6.037)	< 0.001	2.263(1.324 ~ 3.866)	0.003	8.121(4.090 ~ 13.262)	<0.001	2.100(1.102 ~ 3.528)	<0.001	8.129(4.113 ~ 13.716)	<0.001	2.107(1.109 ~ 3.511)	<0.001
Vascular bundle sign	1.199(0.810 ~ 1.776)	0.364	——	——	1.134(0.742 ~ 1.735)	0.561	——	——	1.144(0.810 ~ 1.961)	0.472	——	——	1.433(0.997 ~ 2.016)	0.588	——	——
Shape	0.545(0.348 ~ 0.854)	0.008	0.427(0.236 ~ 0.771)	0.005	0.557(0.356 ~ 0.871)	0.01	0.579(0.311 ~ 1.075)	0.084	1.617(1.153 ~ 2.644)	0.013	0.493(0.281 ~ 0.867)	0.003	1.800(1.204 ~ 2.870)	0.024	0.498(0.287 ~ 0.881)	0.006
Boundary	0.539(0.399 ~ 0.879	0.009	0.404(0.232 ~ 0.704)	0.001	0.603(0.408 ~ 0.893)	0.012	0.346(0.194 ~ 0.616)	<0.001	0.622(0.420 ~ 0.924)	0.017	0.422(0.210 ~ 0.614)	<0.001	0.633(0.427 ~ 0.933)	0.019	0.426(0.214 ~ 0.626)	0.002
Margin	0.413(0.290 ~ 0.587)	<0.001	0.665(0.402 ~ 1.100)	0.112	0.411(0.290 ~ 0.583)	<0.001	0.661(0.391 ~ 1.119)	0.123	0.414(0.291 ~ 0.588)	<0.001	0.716(0.462 ~ 1.148)	0.152	0.420(0.300 ~ 0.594)	<0.001	0.723(0.466 ~ 1.157)	0.161
Lobulation	13.140(5.260 ~ 32.828)	<0.001	5.939(2.709 ~ 13.019)	≤ 0.001	16.361(8.840 ~ 30.279)	<0.001	11.949(5.897 ~ 24.211)	<0.001	6.042(2.196 ~ 19.304)	0.002	2.614(0.880 ~ 9.104)	<0.001	8.014(2.267 ~ 21.077)	0.007	2.619(0.888 ~ 9.119)	0.001
Spiculate	6.807(3.081 ~ 15.038)	<0.001	2.521(0.994 ~ 6.392)	0.052	5.245(3.134 ~ 8.777)	<0.001	2.465(1.277 ~ 4.757)	0.007	14.877(1.993 ~ 84.277)	0.034	9.747(1.033 ~ 83.144)	0.047	17.012(4.133 ~ 81.277)	0.047	9.749(1.039 ~ 81.167)	0.049
Nodule diameter	1.447(1.314 ~ 1.593)	<0.001	0.55(0.422 ~ 0.717)	≤ 0.001	1.472(1.341 ~ 1.616)	<0.001	0.440(0.330 ~ 0.588)	<0.001	1.298(1.216 ~ 1.401)	<0.001	0.544(0.491 ~ 0.688)	<0.001	1.299(1.219 ~ 1.441)	<0.001	0.547(0.493 ~ 0.689)	<0.001
Solid component diameter	1.678(1.524 ~ 1.848)	<0.001	2.019(1.668 ~ 2.446)	≤ 0.001	1.654(1.510 ~ 1.811)	<0.001	2.074(1.679 ~ 2.564)	<0.001	1.645(1.275 ~ 1.895)	<0.001	1.666(1.412 ~ 2.098)	<0.001	1.651(1.279 ~ 1.910)	<0.001	1.668(1.414 ~ 2.102)	<0.001
Area	1.037 (1.028-1.046)	<0.001	1.025 (1.013-1.037)	<0.001	1.041 (1.032-1.050)	<0.001	1.029 (1.006-1.052)	0.015	1.038 (1.029-1.047)	<0.001	1.024 (1.012-1.036)	<0.001	1.039 (1.030-1.048)	<0.001	1.026 (1.014-1.038)	<0.001

Supplementary Table S2. VIF for predictors in the final 10 mm MPVR model.

Predictor Variable	VIF
Pleural Indentation	1.18
Shape	1.32
Boundary	1.55
Lobulation	1.49
Spiculation	2.41
Nodule diameter	2.38
Solid component diameter	1.92
Area	1.32

VIF, Variance Inflation Factor.

Supplementary Table S3. Pairwise DeLong *p*-values for model AUCs.

Model	Lung Window	2 mm MPVR	4 mm MPVR	6 mm MPVR	8 mm MPVR	10 mm MPVR	12 mm MPVR	14 mm MPVR
Lung Window	—	0.21	0.098	0.065	0.072	<0.001	0.055	0.089
2 mm MPVR	0.21	—	0.55	0.004	0.005	<0.001	0.48	0.61
4 mm MPVR	0.098	0.55	—	0.028	0.032	0.003	0.72	0.68
6 mm MPVR	0.065	0.004	0.028	—	0.85	0.084	0.009	0.015
8 mm MPVR	0.072	0.005	0.032	0.85	—	0.061	0.012	0.020
10 mm MPVR	<0.001	<0.001	0.003	0.084	0.061	—	0.002	<0.001
12 mm MPVR	0.055	0.48	0.72	0.009	0.012	0.002	—	0.81
14 mm MPVR	0.089	0.61	0.68	0.015	0.020	<0.001	0.81	—

Values represent Holm-adjusted *p*-values from pairwise DeLong's tests.
